# On Cancer

**Published:** 1890-12-06

**Authors:** 


### The Practitioner's Mirror and Retrospect.

[*The Editor will be glad to receive offers of co-operation and contributions from members of the profession. There is no doubt that much valuable material full of interest and instruction is at present lost to science. This is largely so in the case of* (1) *the younger and less-known members of the hospital staff* (2) *those connected with cottage hospitals, and* (3) *the great body of medical practitioners not attached to any institution. The co-operation of all is cordially invited to enable the Editor to make* The Hospital *of the utmost possible use and value to the profession. Specialists willing to review books on their special subjects are invited to communicate this fact at once. All letters should be addressed to* The Editor, The Lodge, Porchester Square, London, W.]

### THE PATHOLOGICAL SOCIETY OF LONDON.

### ON CANCER.

At the meeting on Tuesday, December 2nd, Dr. W. Russell read a paper entitled, "the characteristic organism of cancer." In commencing, he said that for some years past he had been occupied with tracing the modes of cancer growth in various organs. During the course of these investigations he had come across certain structures which he was unable to place under any of the tissue cell modifications. The special method he employed for staining these structures was by fuchsine and then by iodine green. The iodine green replaced the fuchsine in everything except in these structures. It was of these structures which remained stained red by this process that he was about to speak. For laboratory purposes he proposed calling them "fuchsine bodies." The question naturally arose as to whether they were accidental bodies, or if they were the result of some form of degeneration. In appearance they were perfectly round and homogeneous hyaline bodies. In order to determine that they were not the result of tissue degenerations, all the various recognised tissue degenerations were examined by similar processes, and with but few exceptions with only negative results. In simple tumours these bodies were not present, nor were they present in primary syphilitic sores, or in condylomata. They were, however, found in the case of a tumour of the dura mater of a gummatous nature. In one curious case of syphilis which had resisted all treatment they were also found. They were once found in a case of gelatinous degeneration of the knee joint, and this case had old sinuses in connection with the joint. Fuchsine bodies were also found in one case of chronic superficial ulcer, and in a lung affected by tuberculosis, together with a case of mammary adenoma which may have become malignant. He had examined forty-five cases of various forms of cancer taken in the order of occurrence, and of the disease occurring in almost all the organs. In two of these cases the fuchsine bodies were not found; in all the remaining forty-three they were present. The bodies occurred scattered in foci, and they are not necessarily present in every section, or in every part of the tumour. They might be present in the small-celled infiltration at the margins of a cancerous growth, in the connective tissues themselves, or in the epithelial cells and also in the lymphatics. In one case of very diffuse cancer, they were present in the splenic pulp outside the deposits which occurred in this organ. They usually occurred in clusters of two, three, or even twenty or more. They showed a clear space around them, but this space was not seen in the bodies found in the lymphatic vessels. With a lens of 100 diameters, they appeared as perfect spheres, but varying very much in size. They were homogeneous and structureless. The larger clumps were held together by a clear medium. In comparing them with the nucleus of the cancer cells, these last structures also retained the fuchsine stain, but from the nuclei the colour could be readily removed by a bleaching process. The next question discussed was whether they were animal or vegetable, with the conclusion that they were the latter. Reference was then made to the work on the subject which had been done on the Continent, especially in connection with Paget's disease. Some remarks were next made upon some of the lowest forms of animal life, and on the stages in the development of the sporosperms. The fuchsine bodies also stained with methyl violet by Gram's method, and also with logwood and eosine. With regard to the organisms, both the individuals and also the groups were surrounded by clear spheres; with Gram's method the individual might be seen to apparently bud, and if followed to the next stage the body appears with the bud nearly separated, and then a stage in which there was a chain of four bodies just united together. Smaller bodies appeared to be cast off from the larger ones, and to enter the cells in the neighbourhood, producing the following effects, namely, the protoplasm of the cell became clear and the nucleus disappeared, so that finally the fuchsine body remained in the centre of a clear space. The fuchsine body appeared to clarify and, in fact, to liquify the cell protoplasm in which it is lying, so that the capsule surrounding the body was only an apparent one. In conclusion, he believed we had to do with a fungus, and one which belonged to the same class as the yeast fungus. From its nature it implies the formation of fermention products, which produce changes in the tissues, the magnitude of which cannot be disproportionate to the anatomical changes present in cancer.

Mr. Roger Williams said we must first know more of the nature of the organisms in the normal animal before we could be in a position to judge of the significance of any given organism found in any given case, and especially so with regard to cancer. Facts pointed strongly to the conclusion that cancer was not a disease the nature of which could be produced by an organism. In other words, cancer was an organised body, and as such it was difficult to imagine how it could be due to an organism.

Mr. Shattock referred at some length to the experiments he had made in conjunction with Mr. Ballance and said that in their communication published about a year ago, they had described similar bodies to those just described by Dr. Russell. They never succeeded in making cultivations of any organism from cancer. Although they had failed to find a micro-organism, they had nevertheless suggested the possibility of a protozoon being present. The bodies figured and described by them were in every way similar to those described by Dr. Russell and they had even noticed the appearance of budding and had suggested the name of "carcinozoa" for the bodies. They were now experimenting by inoculating sporosperms into rabbits' ears and had, in some cases, produced some nodules, the nature of which they were as yet unable to state. Dr. Russell in replying said that he had brought the work forward in order to have it put to the test, but he must say that the organism he had described, had no connection whatever with the appearances figured by Messrs. Ballance and Shattock, that is to say, to their chromogenic bodies, and the methods of staining were very different in the two cases.

